# NG2 antigen is a therapeutic target for *MLL*-rearranged B-cell acute lymphoblastic leukemia

**DOI:** 10.1038/s41375-018-0353-0

**Published:** 2019-01-11

**Authors:** Belen Lopez-Millan, Diego Sanchéz-Martínez, Heleia Roca-Ho, Francisco Gutiérrez-Agüera, Oscar Molina, Rafael Diaz de la Guardia, Raúl Torres-Ruiz, Jose Luís Fuster, Paola Ballerini, Ute Suessbier, Cesar Nombela-Arrieta, Clara Bueno, Pablo Menéndez

**Affiliations:** 1 Department of Biomedicine, School of Medicine, Josep Carreras Leukemia Research Institute, University of Barcelona, Barcelona, Spain; 2 Molecular Cytogenetics Group, Human Cancer Genetics Program, Centro Nacional de Investigaciones Oncológicas (CNIO), Madrid, Spain; 3 Pediatric Hematology and Oncology Section, Hospital Clínico Virgen de la Arrixaca, Murcia, Spain; 4 Pediatric Hematology, Armand Trousseau Hospital, Paris, France; 5 Hematology Department, University Hospital-University of Zurich, Zurich, Switzerland; 6 Centro de Investigacion Biomedica en Red-Oncología (CIBERONC), Zurich, Switzerland; 7 Instituciò Catalana de Recerca i Estudis Avançats (ICREA), Barcelona, Spain

**Keywords:** Acute lymphocytic leukaemia, Acute lymphocytic leukaemia

## Abstract

B cell acute lymphoblastic leukemia (B-ALL) is the most common childhood cancer, with cure rates of ∼80%. MLL-rearranged (MLLr) B-ALL (MLLr-B-ALL) has, however, an unfavorable prognosis with common therapy refractoriness and early relapse, and therefore new therapeutic targets are needed for relapsed/refractory MLLr-B-ALL. MLLr leukemias are characterized by the specific expression of chondroitin sulfate proteoglycan-4, also known as neuron-glial antigen-2 (NG2). NG2 was recently shown involved in leukemia invasiveness and central nervous system infiltration in MLLr-B-ALL, and correlated with lower event-free survival (EFS). We here hypothesized that blocking NG2 may synergize with established induction therapy for B-ALL based on vincristine, glucocorticoids, and l-asparaginase (VxL). Using robust patient-derived xenograft (PDX) models, we found that NG2 is crucial for MLLr-B-ALL engraftment upon intravenous (i.v.) transplantation. In vivo blockade of NG2 using either chondroitinase-ABC or an anti-NG2-specific monoclonal antibody (MoAb) resulted in a significant mobilization of MLLr-B-ALL blasts from bone marrow (BM) to peripheral blood (PB) as demonstrated by cytometric and 3D confocal imaging analysis. When combined with either NG2 antagonist, VxL treatment achieved higher rates of complete remission, and consequently higher EFS and delayed time to relapse. Mechanistically, anti-NG2 MoAb induces neither antibody-dependent cell-mediated not complement-dependent cytotoxicity. NG2 blockade rather overrides BM stroma-mediated chemoprotection through PB mobilization of MLLr-B-ALL blasts, thus becoming more accessible to chemotherapy. We provide a proof of concept for NG2 as a therapeutic target for MLLr-B-ALL.

## Introduction

B cell acute lymphoblastic leukemia (B-ALL) is the most common childhood cancer [1]. Over the past 20 years, progress in molecular diagnosis, patient stratification, disease biology, therapy, and supportive care has considerably improved disease management and clinical outcome. The backbone of current induction or re-induction post-relapse treatment protocols is vincristine, glucocorticoids, and l-asparaginase (VxL), with or without an anthracycline [2], resulting in ∼90% of children entering complete remission (CR) [3] and eventual cure rates of ∼80% [4, 5].

Despite the overall progress in treatment, relapsed B-ALL is the fifth most prevalent pediatric cancer, and B-ALL remains the most common cause of death from malignancy in children [6, 7]. Moreover, several B-ALL cytogenetic–molecular subgroups remain high risk, with 5-year event-free survival (EFS) and overall survival (OS) rates <30% [8–10]. B-ALL carrying MLL rearrangements (MLLr-B-ALL), particularly the t(4;11)+/MLL-AF4+ B-ALL, is of special interest because of its dismal prognosis, common therapy refractoriness, and central nervous system (CNS) infiltration [11]. Patients experiencing early relapse or therapy refractoriness have a poor long-term survival and, in these cases, the best therapeutic option is hematopoietic stem cell transplantation (HSCT) following induction into second remission. However, these patients commonly fail to achieve a second remission; moreover, current chemotherapy is associated with morbidity and serious side effects such as infertility, impaired development, and greater risk of secondary neoplasms [12, 13]. Thus, innovative directed/targeted therapeutic approaches are in high demand for high-risk B-ALL and relapsed/refractory (R/R) B-ALL, and targeting leukemia-initiating cells and CNS-infiltrating leukemia cells is key to overcome therapy resistance, relapse, and CNS disease [14, 15].

Chondroitin sulfate proteoglycan-4, also known as neuron-glial antigen-2 (NG2), is a transmembrane proteoglycan barely expressed in normal hematopoietic cells [16, 17]. Conversely, ~90% of 11q23/MLLr leukemias specifically express NG2, which has been incorporated into diagnostic workflows for leukemia immunophenotyping because of its predictive value for MLLr [16, 18–21]. Against this background, we recently reported that NG2 is involved in leukemia invasiveness and CNS infiltration in MLLr-B-ALL, and high levels of NG2 expression in B-ALL blasts correlates with lower EFS, higher numbers of circulating blasts and more frequent CNS disease/relapse. In the present study, we investigated whether NG2 might represent a therapeutic target for MLLr-B-ALL. Using robust preclinical patient-derived xenograft (PDX) models, we show that NG2 antagonists synergize with VxL-based induction therapy, leading to an extensive mobilization of MLLr-B-ALL blasts from bone marrow (BM) into peripheral blood (PB) where they become more accessible/sensitive to VxL-based chemotherapy, resulting in higher CR rates (CRRs), and consequently, higher EFS and delayed time to relapse.

## Methods

### Patient samples and immunophenotyping

Leukemic samples at presentation were used from *n* = 5 independent MLLr-B-ALL patients with complete immunophenotypic and molecular/cytogenetic diagnosis. Four patients were t(4;11)/MLL-AF4+, and one patient was t(1;11)/MLL-EPS15+. Patients’ mononuclear cells with >85% CD45^low^CD19^+^CD34^+^CD10^−^NG2^+^ MLLr blasts were isolated by density gradient centrifugation using Ficoll-Hypaque (Amersham Biosciences, Uppsala, Sweden). MLL/(11q23) status was confirmed by fluorescence in situ hybridization [22]. Blasts were immunophenotyped using the monoclonal antibodies (MoAbs) CD45-FITC, CD19-APC, CD10-PerCP-Cy5.5, CD34-PE-Cy7 (BD Biosciences, San Jose, CA), and NG2^−^PE (Beckman, Barcelona, Spain), and the NG2^+^ and NG2^−^ blast populations were isolated by fluorescence-activated cell sorting (FACS) using a FACSAria cell sorter (BD Biosciences). The Institutional Review Board of the Hospital Clinic of Barcelona approved the study, and all patients’ parents gave written informed consent.

### Drugs and antibodies

Vincristine (Selleckchem, Houston, TX, USA) and dexamethasone (Sigma-Aldrich, St Louis, MO, USA) were reconstituted in dimethyl sulfoxide. l-asparaginase (Kidrolase^®^; EUSA Pharma, Oxford, UK) and chondroitinase-ABC (Ch’ase, Sigma-Aldrich) were reconstituted in phosphate-buffered saline (PBS) according to the supplier’s guidelines. Drugs were stored in aliquots at −20 °C. The clone 9.2.27 MoAb was provided by Abcam (Cambridge, UK). The clone 7.1 MoAb-producing hybridoma was kindly provided by Professor Irwin Bernstein (Fred Hutchinson Cancer Center, Seattle, WA, USA). The anti-NG2 7.1 MoAb was produced and purified using standard methods as previously detailed [18]. All drugs were administered by intraperitoneal (i.p.) injection.

### PDX models, in vivo treatment, and analysis of leukemia engraftment

Eight- to-14-week-old NOD.Cg-PrkdcscidIl2rgtm1Wjl/SzJ mice (NSG; *n* = 116) housed under pathogen-free conditions were used. The Animal Care Committee of The Barcelona Biomedical Research Park approved all experimental procedures with mice (HRH-17-0045-P2). A total of 1–2 × 10^5^ iMLLr-B-ALL cells were transplanted intravenously (i.v) into sublethally (2.25 Gy) irradiated mice as described [23]. Leukemia engraftment was monitored through weekly PB analysis, and human grafts were immunophenotyped by flow cytometry using HLA-ABC-FITC combined with the 7.1 MoAb indicated above. When human engraftment in PB was >0.5% (between weeks 6 and 8, depending on the engraftment kinetics of each patient), mice were homogeneously divided into the following treatment groups: (i) control, (ii) VxL alone, (iii) VxL plus Ch’ase, and (iv) VxL plus 7.1 MoAb. Treatment schedules were as follows: vincristine (V, 0.15 mg/kg) once weekly for 2 weeks, dexamethasone (x, 5 mg/kg), and l-asparaginase (L, 1000 U/kg) daily during 5 days for 2 weeks. This standard induction treatment is known as VxL treatment. Ch’ase (0.06 U/mouse) and 7.1 MoAb (10 mg/kg) were administered daily for 7 days (when given alone) or for 14 days (in combination with VxL). BM aspirates were always performed at the beginning and end of each treatment. Minimal residual disease (MRD) was assessed in the BM of each mouse at the completion of the 15-day treatment schedule. CR was defined as the presence of ≤1% leukemic cells in BM (0.1% in PB). Treatment was then stopped and mice were left untreated for 30 further days to follow-up potential relapse by weekly analysis of engraftment in PB. Analysis of EFS, comparing mice treated with VxL vs. VxL plus NG2 blockers, was performed with Kaplan–Meier curves from the end of the induction treatment (day 15) up to day 45. A leukemic engraftment in PB >0.5% was established to define a relapse event. Mice were sacrificed at the end of the experiment.

### In vitro chemoresistance assay

Primary cells were maintained in a humidified atmosphere with 5% CO_2_ at 37 °C. MLLr-B-ALL primary samples cells were cultured in StemSpam medium (Stem Cell Technologies, Vancouver, BC, Canada) supplemented with stem cell factor, FLT3 ligand, interleukin-3 (IL-3) and IL-7 (all from PeproTech, Rocky Hill, NJ, USA) as described [24]. Bone marrow-derived mesenchymal stromal cells (BM-MSCs) were obtained, grown, and characterized as extensively described by our group [25–27]. A total of 1 × 10^5^ MLLr-B-ALL blasts were co-cultured in a 96-well plate with/without 2 × 10^4^ irradiated BM-MSCs for either 30 min or 24 h. Cells were then exposed to 0.5 or 50 µM VxL for 40 h. Viability (apoptosis) of CD19^+^ B-ALL blasts was measured using 7-AAD on a FACSCanto-II cytometer running FACSDiva software (BD Biosciences) as previously described [28].

### ADCC and CDC assays

Antibody-dependent cell-mediated (ADCC) and complement-dependent cytotoxicity (CDC) assays were performed to directly address the anti-NG2-mediated cytotoxicity, as previously described [29, 30]. Briefly, target cells (SEM (NG2^+^CD20^−^) and Daudi (NG2-CD20^+^)) were pre-incubated with anti-NG2 (7.1 MoAb) or anti-CD20 (Rituximab) MoAb at 10 μg/mL for 30 min at 37 °C. Then, they were incubated with either peripheral blood mononuclear cells (PBMCs) at 10:1 effector:target ratio or 10% human AB serum to induce ADCC and CDC, respectively. Cytotoxicity was determined 4 and 24 h later by analyzing the proportion of AnnexinV+/7-ADD+ by FACS. PBMCs were labeled with 3 µM eFluor to distinguish them from target cells. Specific cell death was determined relative to basal cell death in the absence of MoAb (consistently <5%).

### 3D confocal microscopy of BM

3D imaging of mouse femoral BM cavities was performed as previously described [31]. Briefly, bones were collected, fixed for 6 h in PBS/2% paraformaldehyde (PFA) at 4 °C, and dehydrated by incubation in PBS/30% sucrose for 72 h. Bones were then embedded in OCT, and snap frozen in liquid nitrogen. Thick slices (400–700 µm) of BM were obtained by iteratively sectioning both sides of the femur using a cryostat until the BM cavity was bilaterally exposed. The remaining OCT embedding medium was removed by washing BM slices three times in PBS for 5 min, followed by an additional fixation step in 2% PFA for 2 h at 4 °C. Slices were blocked overnight in blocking buffer (0.2% Triton X-100, 1% BSA, 10% Donkey serum in PBS) and incubated with primary antibodies against endomucin (CD105, Santa Cruz Biotechnology, Dallas, TX, USA), huCD45, and collagen (Abcam) in blocking solution for 72 h at 4 °C. After repeated washing in PBS (3 × 2 h) slices were incubated with a secondary antibody cocktail solution (Jackson ImmunoResearch (Cambridge, UK and Invitrogen) including 2 µm 4′,6-diamidino-2-phenylindole (DAPI) for an additional 72 h at 4 °C. After successive washing steps, immunostained slices were optically cleared through overnight incubation in RapiClear 1.52 (Sunjinlab, Hsinchu City, Taiwan). The slices were then mounted on glass slides for imaging using a SP8 Leica confocal microscope equipped with hybrid detectors using ×10 (HCX-PL-FLUOTAR) and ×20 (HC-PL-APO-CS2) objectives. Imaging data were analyzed and videos assembled using the Imaris v8.2 software (Bitplane AG). Supplemental videos may be found in Supplementary data available with the online version of this article.

### Statistical analysis

Data are expressed as mean ± SEM of independent experiments unless otherwise specified. Statistical comparisons were performed using either paired or unpaired Student’s *t* test, as appropriate. For MRD studies, data are expressed as median (range) and significant differences were analyzed by the median test. CRRs were statistically compared using Pearson’s *χ*^2^ test and EFS curves were compared using the log-rank test. Analyses were performed using GraphPad Prism v6.0 (GraphPad Software Inc., La Jolla, CA, USA) or SPSS software (SPSS version 22, Chicago, IL, USA). Statistical significance was defined as a *p* value < 0.05.

## Results

### NG2 blockage does not elicit cytotoxicity of MLLr-B-ALL blasts but results in in vivo robust blast mobilization into PB

**Fig. 1:**
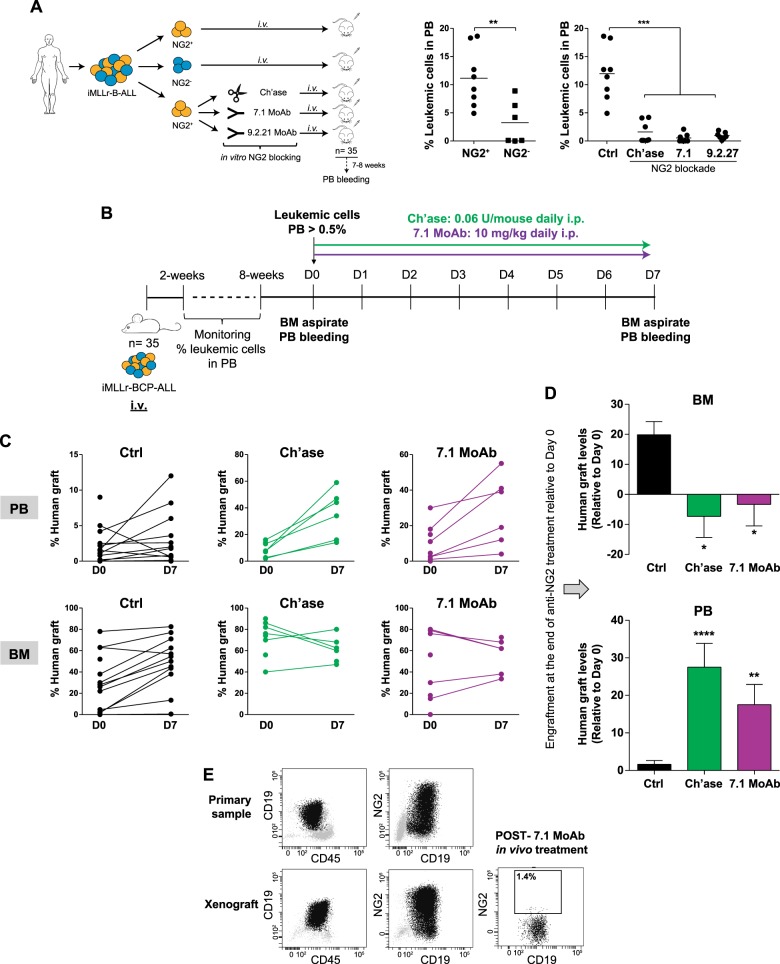
In vivo blockade of NG2 results in the robust mobilization of MLLr-B-ALL blasts into PB. **a** Left, experimental design of the in vitro treatment with NG2 antagonists. Middle, engraftment capacity of NG2^+^ or NG2^−^ MLLr-B-ALL blasts 8 weeks after i.v. transplantation. Right, overnight in vitro exposure of NG2^+^ blasts to the NG2 antagonists Ch’ase, 7.1 MoAb, or 9.2.27 MoAb abolishes their engraftment potential [18]. **b** Experimental design of the in vivo treatment with NG2 antagonists. **c** Monitoring of the levels of leukemic grafts in PB and BM before (day 0) and after (day 7) the indicated treatments. Each line represents the same mouse before and after treatment. **d** Levels of leukemic grafts in BM (top panel) and PB (bottom panel) after the indicated in vivo treatments. Results are shown as mean ± SEM, relative to day 0 (before treatment). **e** Representative FACS plots showing the identical leukemia NG2^+^ phenotype in both diagnostic samples and primografts. The right panel shows the in vivo effectiveness of the 7.1 MoAb, which abolishes NG2 expression in blasts recovered from primografts. *n*, 8–12 mice/group from three different patients. *p < 0.05; **p < 0.01; ****p < 0.0001

We first interrogated the ability of FACS-sorted NG2^+^ and NG2^−^ diagnostic leukemic blasts to reproduce the B-ALL phenotype by engrafting in NSG mice. When equal numbers of NG2^+^ and NG2^−^ B-ALL primary blasts (10^5^ cells/patient, *n* = 4) were intravenously (i.v.) infused into NSG mice, engraftment levels after 8 weeks were 3.5-fold higher in the PB of mice transplanted with NG2^+^ than in equivalent mice transplanted with NG2^−^ blasts (11.2 ± 1.8% vs. 3.2 ± 1.5%, *p* = 0.008) (Fig. 1a, left and middle panels). Overnight in vitro treatment of NG2^+^ B-ALL blasts with either Ch’ase (0.1U/ml) or anti-NG2 MoAb (either 7.1 (0.7 µg/ml) or 9.2.27 (100 µg/ml) clone) abolished their engraftment after i.v. transplantation (12 ± 1.7% vs. <2%, *p* < 0.001) (Fig. 1a, right panel). Importantly, clinical data from MLLr-BCP-ALL infants (*n* = 55) uniformly enrolled in the Interfant treatment protocol reveals that patients with refractory or relapsed disease initially had at diagnosis 28% more NG2-expressing blasts than those patients not experiencing relapse (53% vs. 41%, *p* > 0.05). This suggests that NG2 plays a role in vivo in the propagation of MLLr-B-ALL cells.

**Fig. 2:**
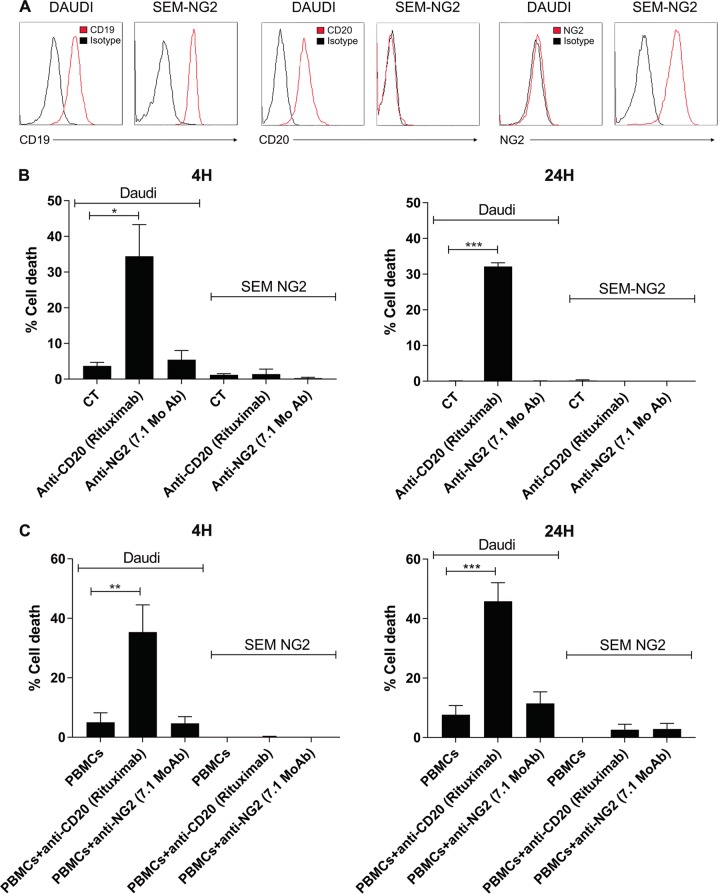
Anti-NG2 MoAb lacks both cellular-mediated (ADCC) and complement-mediated (CDC) cytotoxicity. **a** CD19, CD20, and NG2 surface immunophenotype in Daudi (control) and SEM-NG2 cells. **b**, **c** CDC (**b**) and ADCC (**c**) assays at 4 h (left panels) and 24 h (right panels) with Daudi and SEM-NG2 cells using both anti-NG2 (7.1 MoAb) and anti-CD20 (Rituximab) (*n* = 2). CT: no MoAb control. Human AB serum was used for CDC and human PBMCs were used for ADCC. *p < 0.05; **p < 0.01; ***p < 0.001

We then assessed in vitro and in vivo whether anti-NG2 (7.1 MoAb) effectively eliminates NG2^+^ MLLr-B-ALL blasts. In vitro, anti-NG2 treatment was unable to activate effector mechanisms including CDC (Fig. 2a, b) and ADCC (Fig. 2c), resulting in no cytotoxicity of NG2-expressing MLLr-B-ALL cells in 4- and 24-h assays. As a control, Rituximab (anti-CD20 MoAb) effectively activated both CDC and ADCC cytotoxicity mechanisms to eliminate Daudi cells (Fig. 2). We further addressed whether NG2 blockage elicits cytotoxicity in vivo, thus impacting the engraftment dynamics of MLLr-B-ALL blasts in PDX models (Fig. 1b). A total of 10^5^ NG2^+^ MLLr-B-ALL cells (*n* = 4 patients) were i.v. transplanted and, once PB engraftment reached >0.5%, mice were daily i.p. treated with the NG2 antagonist Ch’ase (0.06 U/mouse) or anti-NG2 7.1 MoAb (10 mg/kg/mouse) for 7 days (Fig. 1b). In line with the in vitro data, NG2 blockage in vivo failed to reduce tumor burden (Fig. 1c). However, when we compared vehicle-treated primografts with primografts treated with either NG2 antagonist after completion of the treatment, we found that treatment with NG2 antagonists significantly reduced leukemic burden in BM that was accompanied by a massive leukemia infiltration in PB (*p* < 0.01; Fig. 1c, d). Importantly, PDX models reproduced the immunophenotype of the de novo primary leukemia, and blasts recovered from primografts treated with NG2 blockers were mainly NG2^−^ (Fig. 1e). These results show that blocking NG2 in vivo mobilizes MLLr-B-ALL blasts to PB. This coupled to our previous data [18], reporting similar engraftment levels of NG2^+^ MLLr-B-ALL blasts when directly delivered intra-BM, suggest that NG2 regulates mobilization to and engraftment in the BM.

**Fig. 3:**
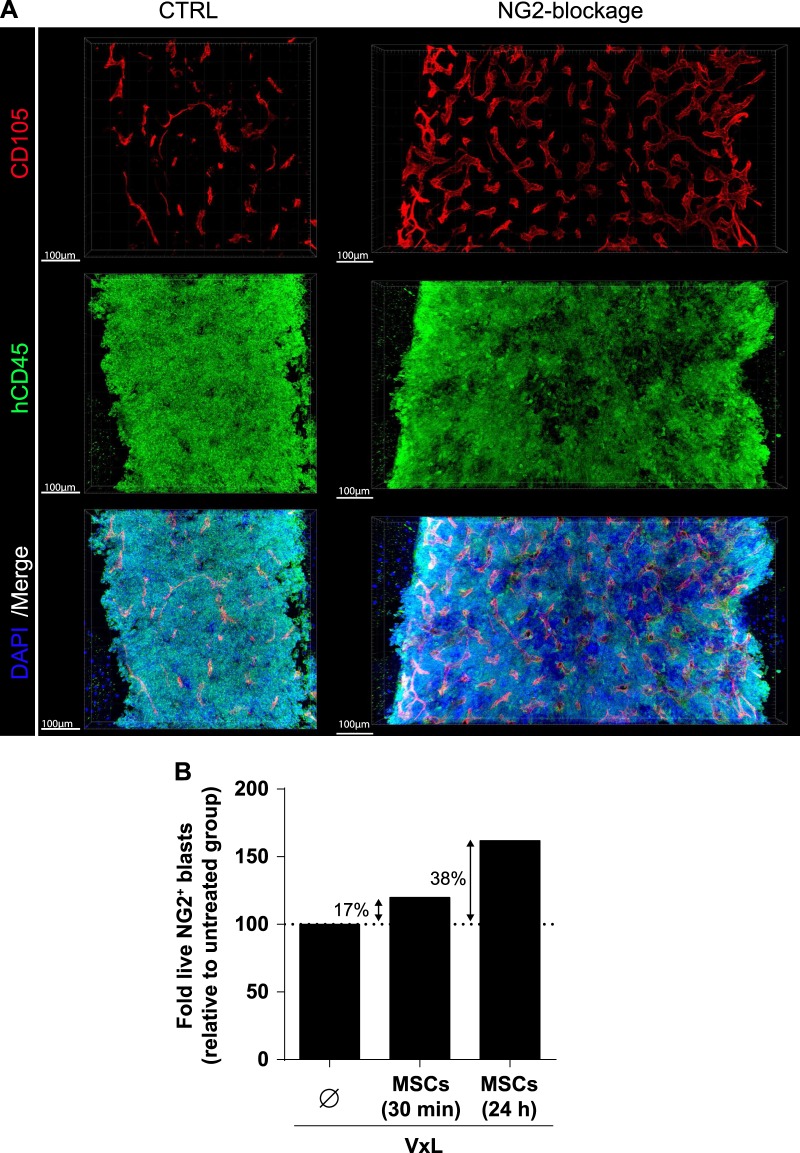
**a** In situ 3D microscopy imaging of BM from primografts treated with NG2 antagonists. Representative images of large tissue volumes from the femoral diaphysis of transplanted mice treated with the vehicle (CTRL, left panels), or with 7.1 MoAb for 7 days (NG2 blockage, right panels). Large DAPI^+^ tissue regions depleted of huCD45^+^ cells are only observed in the BMs from mice treated with 7.1 MoAb. See also Supplementary Videos 1 and 2. **b** BM-MSCs protect MLLr-B-ALL cells against VxL chemotherapy (*n* = 2)

In order to confirm FACS-based quantifications, we employed advanced protocols for in situ 3D BM imaging, which allows to visualize the presence and distribution of the human graft within the native BM microenvironment of control and anti-NG2-treated mice at the end of treatment. Engrafted human B-ALL cells were labeled by immunostaining against huCD45 (Fig. 3a). Consistent with our FACS data, we repeatedly observed the presence of large tissue volumes devoid of human cells, but filled with murine hematopoietic cells in the BM of anti-NG2-treated mice. These patches devoid of huCD45+ cells were substantially smaller and almost absent in control BM (Fig. 3a, Suppl Videos 1 and 2), thereby confirming that NG2 blockage efficiently induces egress and mobilization of BM MLLr-B-ALL leukemic cells.

### NG2 antagonists synergize with VxL therapy and render higher CR rates and EFS in preclinical PDX models of MLLr-B-ALL

**Fig. 4:**
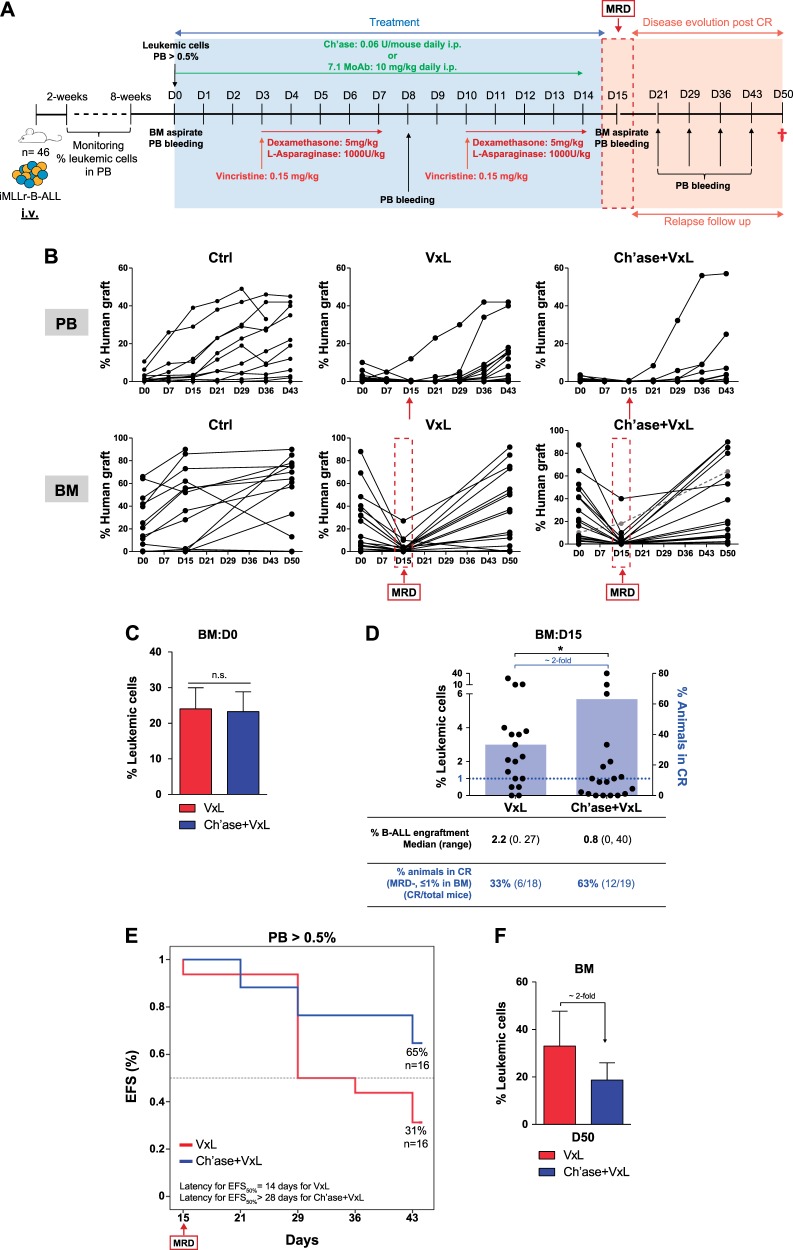
In vivo treatment with Ch’ase sensitizes blasts to VxL, rendering higher CR rates and higher EFS in preclinical PDX models of MLLr-B-ALL. **a** Complete experimental design of the preclinical PDX models detailing in vivo treatments with VxL and NG2 antagonists. After 2 complete cycles of VxL chemotherapy, MRD/CR was evaluated and relapse was followed-up for up to 30 days. **b** Monitoring of the levels of leukemic engraftment in PB and BM for the indicated treatments. PB engraftment was analyzed weekly. BM leukemic engraftment was analyzed using BM aspirates at the end of VxL ± Ch’ase treatment (day 15) and at the end of the follow-up period (day 50). Each line represents the same mouse before and after treatment. **c** Levels of leukemia engraftment in BM at treatment initiation (day 0) for VxL or VxL + Ch’ase mice cohorts. **d** BM levels of MRD at the end of two cycles of VxL ± Ch’ase (day 15). Each dot represents a single mouse. A mouse is considered in CR when the % of blasts in BM <1% (blue horizontal dotted line). The light blue bars represent the proportion of mice in CR (right *Y*-axis) for VxL and VxL + Ch’ase (*n* = 18 mice/group). **e** Kaplan–Meier survival curves for 45 days EFS (*n* = 16 mice). **f** Leukemic burden in BM at sacrifice. *p < 0.05; n.s.: no significant differences

**Fig. 5:**
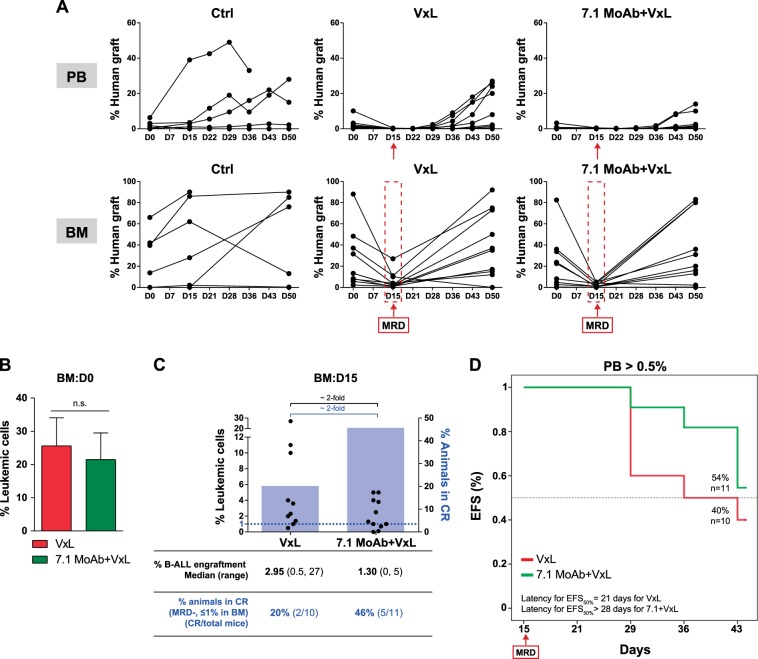
In vivo treatment with 7.1 MoAb sensitizes blasts to VxL, rendering higher CR rates and higher EFS in preclinical PDX models of MLLr-B-ALL. **a** Monitoring levels of leukemic grafts in PB and BM for the indicated treatments. PB engraftment was analyzed weekly. BM leukemic engraftment was analyzed using BM aspirates at the end of VxL ± 7.1 MoAb treatment (day 15) and at the end of follow-up period (day 50). Each line represents the same mouse before and after treatment. **b** Levels of leukemia engraftment in BM at treatment initiation (day 0) for VxL or VxL ± 7.1 MoAb mice cohorts. **c** BM levels of MRD at the end of two cycles of VxL ± 7.1 MoAb (day 15). Each dot represents a single mouse. A mouse is considered in CR when the % of blasts in BM <1% (blue horizontal dotted line). The light blue bars represent the proportion of mice in CR (right *Y*-axis) for VxL and VxL ± 7.1 MoAb. **d** Kaplan–Meier survival curves for 45 days EFS (*n* = 10–11 mice in each group). *p < 0.05; n.s.: no significant differences

**Fig. 6:**
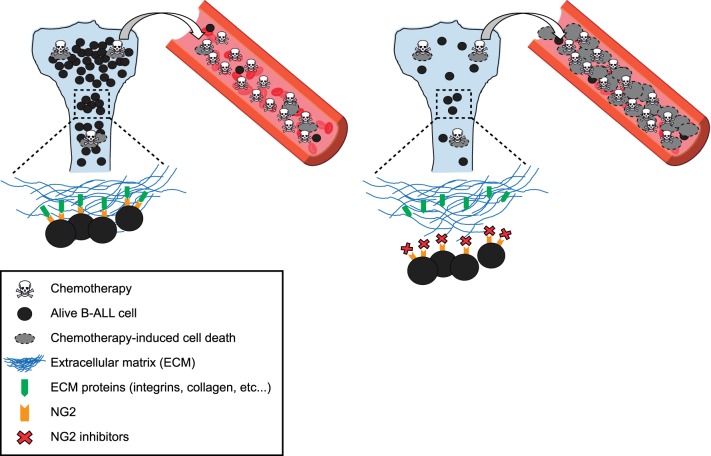
A proposed mechanism of action for NG2 antagonists. NG2 is well established to bind to common components of the extracellular matrix including collagen [40, 41, 57]. This supports a model in which in vivo blockade of NG2 may disrupt the interaction between MLLr-B-ALL blasts and BM stroma, promoting the mobilization of MLLr-B-ALL blasts into PB, thus overriding BM stroma-mediated chemoprotection and making leukemic cells more accessible (sensitive) to conventional chemotherapy

BM stroma confers chemoresistance to leukemic cells in a variety of hematological malignancies [32, 33]. We therefore first tested in vitro whether BM-MSCs also protect NG2^+^ MLLr-B-ALL primary blasts from VxL. NG2^+^ blasts pre-exposed to BM-MSC for 30 min or 24 h displayed ∼20% or ∼40% increased resistance to VxL, respectively, indicating a BM stroma-mediated chemoprotection of MLLr-B-ALL blasts to induction therapy (Fig. 3b). Mobilization of leukemic cells from BM to PB is clinically desirable because circulating blasts become more accessible (and sensitive) to cytotoxic treatments due, in part, to their detachment from the chemoprotective BM niche [34–36]. Therefore, we next tested whether mobilization of MLLr-B-ALL blasts to PB with the NG2 antagonist Ch’ase (Fig. 4) or 7.1 MoAb (Fig. 5) synergizes with VxL treatment in PDX models of MLLr-B-ALL. To do this, engrafted PDXs were treated with vehicle, VxL alone, VxL plus Ch’ase, or VxL plus 7.1 MoAb, and CR (MDR <1% leukemic cells in BM (approx. <0.1% in PB) was assessed at completion of the treatment (day 15)). Mice were then left untreated for 30 days and relapse and EFS were determined (Fig. 4a). In contrast to vehicle-treated mice, VxL treatment was highly efficient and engrafted PDXs treated with two cycles of VxL (14 days) showed a massive decrease in leukemia burden in both BM and PB (Figs. 4b, 5a). Moreover, despite comparable engraftment levels (∼24%) in BM at the time of treatment initiation (day 0) (Figs. 4c, 5b), the levels of B-ALL engraftment in BM at the end of treatment (day 15) was ∼3-fold lower when either Ch’ase (0.8% vs. 2.2%, Fig. 4b, d) or 7.1 MoAb (1.3% vs. 2.95%, Fig. 5a, c) were co-administered with VxL. As a consequence, the rate of animals achieving CR increased 2-fold when NG2 antagonists were co-administered with VxL: 63% vs. 33% for Ch’ase (Fig. 4d) and 46% vs. 20% for 7.1 MoAb (Fig. 5c). To further assess the potential clinical impact of the lower levels of MRD and higher CR rates, treatment was removed and PDXs were followed-up for a 30-day period. Importantly, 45-day EFS was higher in the group treated with either Ch’ase (65% vs. 31%, Fig. 4e) or 7.1 MoAb (55% vs. 40%, Fig. 5d) than with VxL alone. Accordingly, at the time of sacrifice, mice in the VxL-alone group had 50% higher leukemic burden than those receiving VxL combined with NG2 antagonists (Fig. 4f). Collectively, these results indicate that NG2 blockade overrides BM stroma-mediated chemoprotection through PB mobilization of MLLr-B-ALL blasts, which consequently are more accessible to conventional chemotherapy (Fig. 6).

## Discussion

There has been considerable progress in the past two decades in understanding the biology of B-ALL, which together with better molecular diagnosis, patient stratification, and therapy, has substantially improved clinical outcomes. VxL, with or without an anthracycline [2], is currently the backbone of induction (or re-induction post-relapse) treatment protocols with ∼90% of children entering CR [3] and an eventual 5-year cure rate of ∼80% [4, 5]. Nevertheless, relapsed or refractory B-ALL (R/R B-ALL) is still common and remains non-curable [6, 7]. Moreover, age <1 year and MLLr are two major adverse prognostic factors in B-ALL. Accordingly, MLLr-B-ALL patients remain high-risk with 5-year EFS and OS rates <30% [8–10]. The t(4;11)+/MLL-AF4+ B-ALL is of special interest owing to its extremely short latency and dismal prognosis, common therapy refractoriness, and CNS involvement [11]. In R/R B-ALL patients, the best therapeutic options are currently: (i) HSCT following induction into second CR, but they frequently fail to achieve a second remission; or (ii) anti-CD19-targeted immunotherapies, which have shown very promising outcomes although disease-free remissions are usually maintained for limited time [37–39]. Furthermore, current chemotherapy is associated with morbidity and serious side effects such as infertility, impaired development, and greater risk of secondary neoplasms [12, 13]. Thus, new targeted therapeutic approaches are urgently needed for de novo high-risk B-ALL and R/R B-ALL.

Here we analyzed whether NG2, a surface antigen exclusively expressed in 11q23/MLLr leukemias, represents a target for MLLr-B-ALL. The therapeutic interest in targeting NG2 in MLLr R/R B-ALL is based on recent findings involving NG2 in leukemia invasiveness, CNS infiltration, and clinical outcome. Indeed, high expression of NG2 in B-ALL blasts has been associated with lower EFS, hyperleukocytosis, and more common relapse [18]. Our robust preclinical PDX models coupled to in situ 3D confocal imaging of native BM revealed that NG2 is crucial for MLLr-B-ALL engraftment upon i.v. transplantation, and that in vivo blockade of NG2 using either a specific inhibitor Ch’ase or an anti-NG2-specific antibody results in an extensive mobilization of MLLr-B-ALL blasts from BM to PB. These findings are in line with previous studies implicating NG2 antigen with MLLr leukemia invasiveness and migration [18], and are consistent with the known biology of NG2. Importantly, anti-NG2 MoAb does not activate either ADCC or CDC cytotoxicity pathways, thus resulting in a lack of cytotoxicity in vitro and in vivo, which further supports the role of NG2 in regulating MLLr-B-ALL blast mobilization to and engraftment in the BM.

NG2 is a transmembrane proteoglycan barely expressed in normal hematopoietic cells [16, 17], but specifically expressed in ~90% of MLLr acute leukemias [16, 17]. Accordingly, it has been incorporated into diagnostic workflows for leukemia immunophenotyping due to its predictive value for MLLr [16, 18–21]. NG2 is a single-pass type I transmembrane protein that has a very large extracellular domain and short transmembrane and intracytoplasmic domains [40, 41]. The large extracellular domain consists of three subdomains known as D1–3 that signal upon interaction with multiple extracellular matrix components, including laminins (binding through D1) collagen types II, V, VI, and fibronectin (binding through D2), and with tenascin, galectins, and lectins such as P-selectin and α3β1 integrin (binding through D1) [41–43]. It is therefore likely that the NG2 molecule has an important role in the anchoring of blasts to the BM niche, such that the loss of such a cellular interaction results in detachment of MLLr blasts from the BM and their subsequent mobilization to PB. This scenario is similar to that of molecules commonly employed in clinical practice for the mobilization of either hematopoietic progenitors or acute myeloid leukemia (AML) blasts into PB, such as granulocyte-colony-stimulating factor or plerixafor [44–46].

The interaction of leukemic cells with the BM microenvironment in functional niches is a major mechanism underlying leukemia maintenance by favoring leukemic cell growth and clonal evolution of malignant cells, ultimately resulting in therapy resistance and relapse [47–52]. Thus, PB-mobilized MLLr-B-ALL blasts not only become more accessible but might also be sensitized to conventional chemotherapy by overriding BM stroma-mediated chemoprotection, a major mechanism underlying refractoriness to chemotherapy [53, 54]. Our data further show that MLLr-B-ALL blasts mobilized to PB with NG2 antagonists do in fact become sensitized to conventional chemotherapy (VxL) as demonstrated by lower levels of MRD and therefore higher rates of CR at treatment completion, resulting in higher EFS and delayed time to relapse. Previous preclinical studies have successfully used an anti-NG2 MoAb to significantly reduce both adhesion-induced cell spreading and anchorage-independent growth of melanoma cells [55]. Conversely, ectopic expression of NG2 in NG2-null human melanoma cells led to enhanced tumor spreading, unequivocally implicating NG2 as a major regulator of migration and invasion in melanoma [56]. Of note, both NG2^+^ and NG2^−^ cells are found in engrafted mice, suggesting a NG2 regulation in response to a homeostatic adaptation of MLLr leukemic cells. This further supports NG2-targeted treatment (along with current chemotherapy) as a promising adjuvant therapy to disturb such homeostatic adaptation of MLLr blasts.

This is the first study targeting NG2 in human leukemias and provides a proof-of-concept preclinical model for NG2 as a therapeutic target for MLLr-B-ALL (Fig. 6). Because NG2 is a malleable marker with enhanced expression in blasts infiltrating extramedullar hematopoietic sites [18], we propose that a MoAb-based or chimeric antigen receptor (CAR) T-cell-based immunotherapy approaches will benefit current treatment of de novo or R/R MLLr-B-ALL patients. In addition, over 80% of leukemic blasts in MLLr AML are NG2^+^, suggesting that MLLr AML may also be a candidate high-risk leukemia for NG2 targeting. As a note of caution, we must bear in mind that any new immunotherapy despite being directed against a specific antigen not expressed in healthy tissues/cells could be in the long-term accompanied by potential secondary events, not anticipated a priori. CAR T cells or bispecific monoclonal antibodies-based clinical trials have revealed mechanisms of immune escape and even “on-target off-tumor toxicities” toxicities [57]. Therefore, future preclinical studies and phase I clinical trials will definitively determine the implementation of a NG2-based immunotherapy for MLLr acute leukemias. Although Ch’ase and 7.1 MoAb render a very similar effect, a strategy based on a MoAb is expected to display less on-target off-tumor toxicity.

## Supplementary information

Supplementary_Video1

Supplementary_Video2
